# Predicting Depth from Single RGB Images with Pyramidal Three-Streamed Networks

**DOI:** 10.3390/s19030667

**Published:** 2019-02-06

**Authors:** Songnan Chen, Mengxia Tang, Jiangming Kan

**Affiliations:** 1 School of Technology, Beijing Forestry University, No. 35 Qinghua East Road, Haidian District, Beijing 100083, China; chensongnan@xyafu.edu.cn (S.C.); mengxiatang@163.com (M.T.); 2 Key Laboratory of State Forestry Administration on Forestry Equipment and Automation, No. 35 Qinghua East Road, Haidian District, Beijing 100083, China

**Keywords:** predicting depth, monocular image, third-streamed network, pyramidal

## Abstract

Predicting depth from a monocular image is an ill-posed and inherently ambiguous issue in computer vision. In this paper, we propose a pyramidal third-streamed network (PTSN) that recovers the depth information using a single given RGB image. PTSN uses pyramidal structure images, which can extract multiresolution features to improve the robustness of the network as the network input. The full connection layer is changed into fully convolutional layers with a new *upconvolution* structure, which reduces the network parameters and computational complexity. We propose a new loss function including scale-invariant, horizontal and vertical gradient loss that not only helps predict the depth values, but also clearly obtains local contours. We evaluate PTSN on the NYU Depth v2 dataset and the experimental results show that our depth predictions have better accuracy than competing methods.

## 1. Introduction

Depth estimation is a fundamental problem in the field of computer vision and graphics. It has become an important part of understanding the geometric relations of three-dimensional scenes, which is widely applied in intelligent robots [1,2], traffic assistance [3], unmanned driving [4], 3D modeling [5,6], target detection and tracking [7,8,9] and so forth. The depth of the image is defined as the distance from the object to the camera. We need to use the various cues or related information contained in the image to recover the distance information from one or more RGB images.

According to the influence of human factors on the depth estimation, we can divide the methods into active estimation and passive estimation [10]. The active method can obtain depth information with higher accuracy, but the high cost of equipment, enormous demand for energy and difficulty in focusing on an object prevent the method from being widely promoted [11]. The passive method includes several techniques that have developed rapidly in the past few years, such as stereoscopic vision theory [12,13], structure-from-motion (SFM) [14], depth-from-focus (DFF) [15] and depth-from-defocus (DFD) [16,17]. The stereo vision method needs to solve the problem of feature extraction and matching that is mainly used for static images. SFM applies camera motion information at different time intervals to estimate the depth. DFF uses the image set of a monocular camera, which is composed of multiple focus parameters, to obtain depth information. However, it requires multiple images and has difficulty meeting real-time requirements.

The purpose of the paper is to predict the depth value of each pixel from a single image, however inferring the underlying depth is an ill-posed and inherently ambiguous problem. Monocular images contain only two-dimensional information, losing the depth information in these scenes. As a result, we cannot directly perceive the distance, size and speed of these objects in monocular images. In addition, objects in some scenes (such as indoor scenes) have considerable changes in texture and structure and there are multiple object occlusion problems, which have a considerable impact on the accurate prediction of depth information.

In this paper, we propose a new method to predict the depth from a single image. We directly regress on the depth using a convolutional neural network (CNN) with three streams: one that first estimates the global structure of the scene, a second that estimates the local structure and a third that estimates the detailed structure. The three streams fuse the convolutional feature into an upsampling architecture. The contribution of our work is as follows. First, the input of the pyramidal third-streamed network (PTSN) model is a series of pyramid images, which are composed of the same view images with multiple resolutions and are convenient for networks to extract the feature map of different scales. Secondly, we use the multiscale network to predict the depth of the monocular image, which can predict the global and local information of the image and retain the edge details. Finally, we train the network by optimizing the loss function by adding the 4-directional gradients of the image based on the L2 and L1 norms. The results show that it is close to the ground truth and also has a similar local structure. Our final depth output achieves a better estimation than what is achieved with other state-of-the-art methods on the NYU Depth v2 dataset [18].

## 2. Related Work

Depth estimation plays an important role in 3D reconstruction, object detection and recognition, semantic segmentation and so forth. It has many applications and we discuss only the monocular method in this paper. The early work on depth prediction focuses on machine learning methods based on prior knowledge and hypothesis. Karsch et al. [19] proposed a nonparametric sampling method to extract depth information from video. However, this model has a long prediction time, poor prediction of outdoor scenes and aerial objects, and to a large extent depends on the collected database. Liu et al. [20] formulated monocular depth estimation as a discrete–continuous optimization problem and obtained the depth by performing inference in a graphical model using particle belief propagation. Saxena et al. [21] assumed that all scenes are horizontally aligned with the ground plane and proposed to predict the depth by training the Markov random field (MRF), which incorporates multiscale local and global image features. They introduced the superpixel concept in the MRF formulation to solve the maximum a posteriori estimation (MAP) problem. However, the MRF model is often difficult to train and the most common approximate methods lack flexibility and require a special scanner to collect data. Subsequently their work was expanded to the reconstruction of 3D scenes [22] and they assumed that the scenes are composed of many small planes, which could predict for objects with nonvertical structures for 3D reconstruction. Inspired by the research of Saxena et al., Liu et al. [23] combined semantic segmentation with depth estimation. The scene is first semantically segmented and then the semantic segmentation scene prediction is merged with the MRF to complete the deep reconstruction. Hoiem et al. [24] did not clearly predict depth; instead the image is divided into several regions according to the geometric structure (horizontal, vertical, etc.) and a 3D model of a simple scene is reconstructed.

Recently, convolutional neural networks (CNNs) have been proved to be highly effective for depth estimation [25,26,27,28,29,30]. Liu et al. [27] proposed combining conditional random fields (CRF) and CNNs to predict the superpixel level depth. The model can maintain the edge and not rely on any geometrical prior and additional information; however the performance on dramatic changes and local details are poor. Roy et al. [28] combined the random forest with CNNs using a regression tree (convolutional regression tree) to process sample data and the single regression result of each convolutional regression tree was merged into the final depth estimation. Li et al. [31] used the deep convolutional network to extract the block feature of different scales of an image and then refined them by the hierarchical CRF.

Other methods have harnessed pretrained CNNs for depth estimation. Eigen et al. [25], for the first time, proposed to regress a dense depth map from a single image using two CNNs: The first being coarse net, which estimated the global structure of the scene and the convolutional layers from Alex-Net [32] and the second being fine net, which refined the depth map of global feature of the coarse network prediction together with the original image. Another study by Eigen et al. [26] addressed three different computer vision tasks using a single multiscale CNN architecture and the number of scales in the network changed from 2 to 3. Laia et al. [30] designed a network structure based on the residual network and a small convolution kernel was used, instead of a large kernel, to realize an *upconvolution* structure. This can save training time and has fewer parameters and less training data. Chakrabarti et al. [33] used a neural network (VGG-19) to approach the problem of monocular depth estimation using a globalization procedure to find a consistent depth map that could match all the local derivative distributions.

The depth camera has also been used to complete the depth estimation, such as Kinect v2 [34]. In the testing phases, our method predicted the depth map from a single RGB image based on the PTSN without a depth camera.

## 3. Methodology

In this section, we describe our model for depth prediction from a single RGB image. First, we propose a PTSN and then augment the training data through random online transformations. Finally, we propose a loss function that achieves better output in our model.

### 3.1. Network Architecture

**Figure 1:**
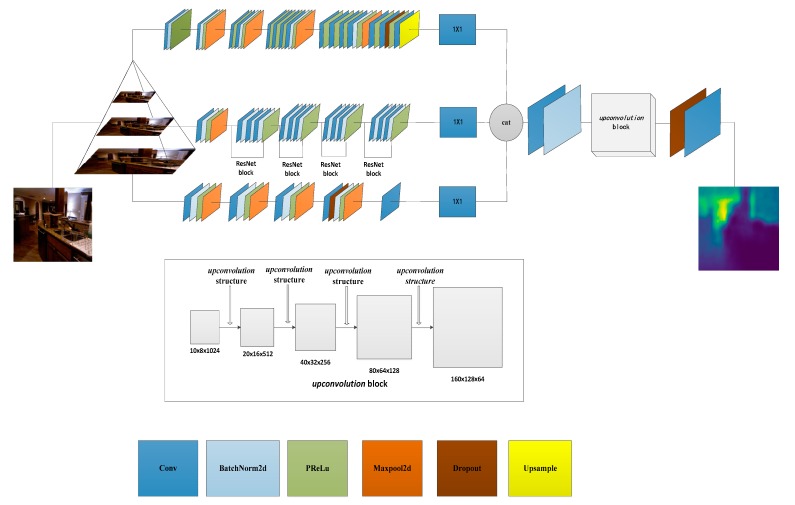
Our three-streamed depth estimation network architecture. Each stream extracts image features at different scales. We used six colors to represent the different operation modules (convolution, normalization, activation, pooling, dropout and upsampling). Cubes are *upconvolution* blocks in gray, which are composed of four *upconvolution* structures (see Figure 2).

Our network consisted of three streams and four novel *upconvolution* structures, as shown in Figure 1. We constructed the three-layer image pyramid structure as the input for the three-stream network to achieve global, local and detailed feature extraction from a single image. The first stream is similar to the VGG-19 network, but we regularized the convolution results of each layer to accelerate deep network training by reducing the internal covariate shift and using a parametric rectified linear unit (PReLU) [35], instead of a rectified linear unit (ReLU), to improve the model’s ability with less computational cost and reduce the risk of overfitting. We set the input of 160×120 pixels in the first stream, removing the last pooling layer and a fully connected layer of VGG-19. The last output layer results are upsampled to 10×8 pixels. The residual learning framework [36] was presented to simplify the training of networks that can be difficult to optimize when the networks have gradually increasing depth. In the second stream, we adopted the ResNet-50 network, inputting sizes of 320×240 pixels to extract the local information. The last full convolution layer outputs 10×8 pixels, meanwhile we removed all the fully connected layers. To deal with high-resolution images, the third stream is composed of one 11×11 convolution, three successive 5×5 convolutions with normalization and one 3×3 convolution to ensure the same size as the other streams. Through the hierarchical image pyramid and three-streamed CNN, we obtained the three dimensions of the scale feature map, where the dimensions are 512, 2018 and 64. In the cascading process of the feature map, if the dimensions are not reduced, the number of channels of the output feature map will increase to 2594 after the serial operation. Too many feature map channels will lead to overfitting the features, so the output is reduced to half in dimension by a 1×1 convolution kernel and the feature fusion is a coarse 10×8×1024 depth map.

**Figure 2:**
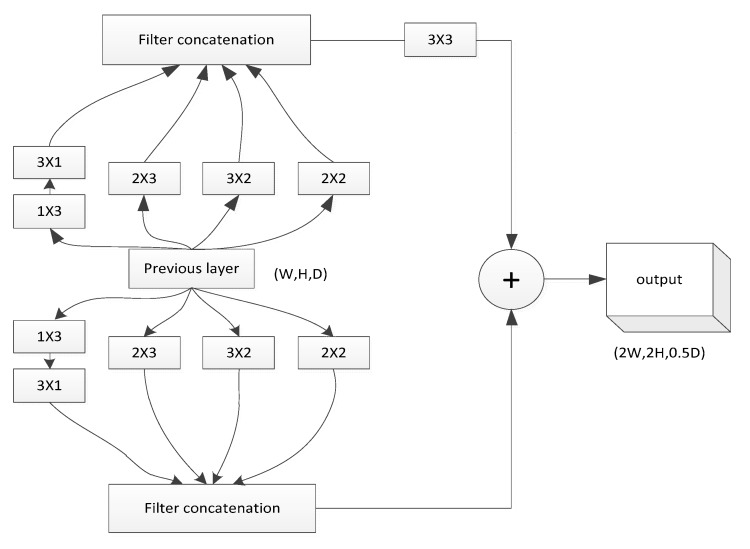
*Upconvolution* structure. This structure is similar to upprojection [30], but in this paper the symmetry convolution is replaced by asymmetric convolution. This version is more efficient and can speed up the process of training. By using this structure, the scale of the feature map is doubled, while the depth value can be reduced by half ([W, H, D]->[2W, 2H, 0.5D]).

The *upconvolution* structure, similar to the fast upprojection block [30], applies the 5×5 convolutions separately on the two branches. However, each convolution kernel is further divided into an asymmetric structure in our paper. This structure is used to greatly reduce the number of parameters and overfitting and accelerate the calculation speed. In addition, we found that this asymmetric convolution structure is more effective than the fast upprojection block [30] during the training time of the whole network. The efficiency increases by approximately 5% and the structure can deal with more and richer spatial features and increase their diversity. Figure 2 shows our *upconvolution* structure. We used it to change the feature map size from 10×8 pixels to 160×128 pixels and the final output resolution is higher than that of Eigen et al. [25,26].

Following the three–stream and four novel *upconvolution* structures, dropout is applied and predicts that the target depth is output by the last layer. The exact network configurations we used in our experiments are shown in Table 1.

### 3.2. Data Augmentation

Whether the training set is sufficient and the particular category of data is sufficient plays a significant role in the process of deep learning. It is a good choice to avoid overfitting and enhance robustness for data augmentation. We trained our network on RGB inputs to predict the corresponding depth map and apply the random offline transformation to augment the training data. Input images and the target ground truth are flipped around the vertical axis and randomly increased brightness, contrasted and multiplied with a random RGB value $c\in {\left[0.8,1.2\right]}^{3}$ to avoid the influence of light.

### 3.3. Loss Function

The loss function is used to measure the degree of disagreement between the predicted value and the ground truth of the model. The most common loss functions for solving regression problems are the L1-norm and L2-norm. However, the L1-norm manifests as non-smooth when the error is close to zero and the disadvantage of the L2-norm is that when outliers exist, these points will be the main components of loss. To avoid these problems, we used the loss function comparing the predicted depth map *p* and ground-truth p^*^, defining the difference between *p* and p^*^ as $d=|p-p^{*}|$, the loss function can be expressed as Equation (1):

(1) $$loss=loss_{data}+\frac{1}{n}\sum_{i}\left[{\left(\nabla_{x}d_{i}\right)}^{2}+{\left(\nabla_{y}d_{i}\right)}^{2}\right]+l_{reg}(w)$$

where *i* is a pixel index to be summed over *n* valid depth pixels. $\nabla_{x}d_{i}$ and $\nabla_{y}d_{i}$ are the horizontal and vertical gradients of the difference between *p* and p^*^, which could reduce the prediction error of the local structure. $loss_{data}$ is a piecewise function, inspired by Laia et al. [30], δ is a constant: 0.2×max(d). We set $loss_{data}$ to:

(2) $$loss_{data}=\{\begin{array}{ll} \frac{1}{2\delta^{2}}d^{2}+\frac{\delta }{2} & d>\delta ,\\ \left|d\right| & d\leq \delta . \end{array}$$

$l_{reg}(w)$ is the penalty term for the loss function to prevent overfitting; we set $l_{reg}(w)$ to:

(3) $$l_{reg}(w)=\frac{\lambda }{2n}\sum_{i=1}^{n}w_{i}^{2}$$

where $w_{i}$ is a parameter learned by the network, λ∈[0,+∞) is the regularization coefficient, $l_{reg}(w)$ enables the learning algorithm to perceive the input with a high variance, so the feature weight with a small covariance between the input and the output target will decrease.

## 4. Experimentation

### 4.1. Dataset

We used the NYU Depth v2 [18] dataset to train our model. The dataset is composed of video sequences of various indoor scenes captured by a Microsoft Kinect camera, which mainly include two parts: one is a subset of the video data accompanied by dense multiclass labels that was preprocessed by filling in missing depth values, the other is the raw RGB, depth and accelerometer data with no preprocessing and must be projected into the RGB coordinate space. The NYU Depth v2 [18] raw dataset consists of 464 scenes, which can be divided into 249 training scenes and 215 testing scenes. We randomly selected approximately 47 K images from different training scenes to build our dataset, the final training set comprises approximately 120 K images after offline data augmentation. The dataset we use for training is significantly smaller than the related work needed in [25,26]. We evaluated the PTSN on the 654 NYU Depth v2 [18] testing images.

**Figure 3:**
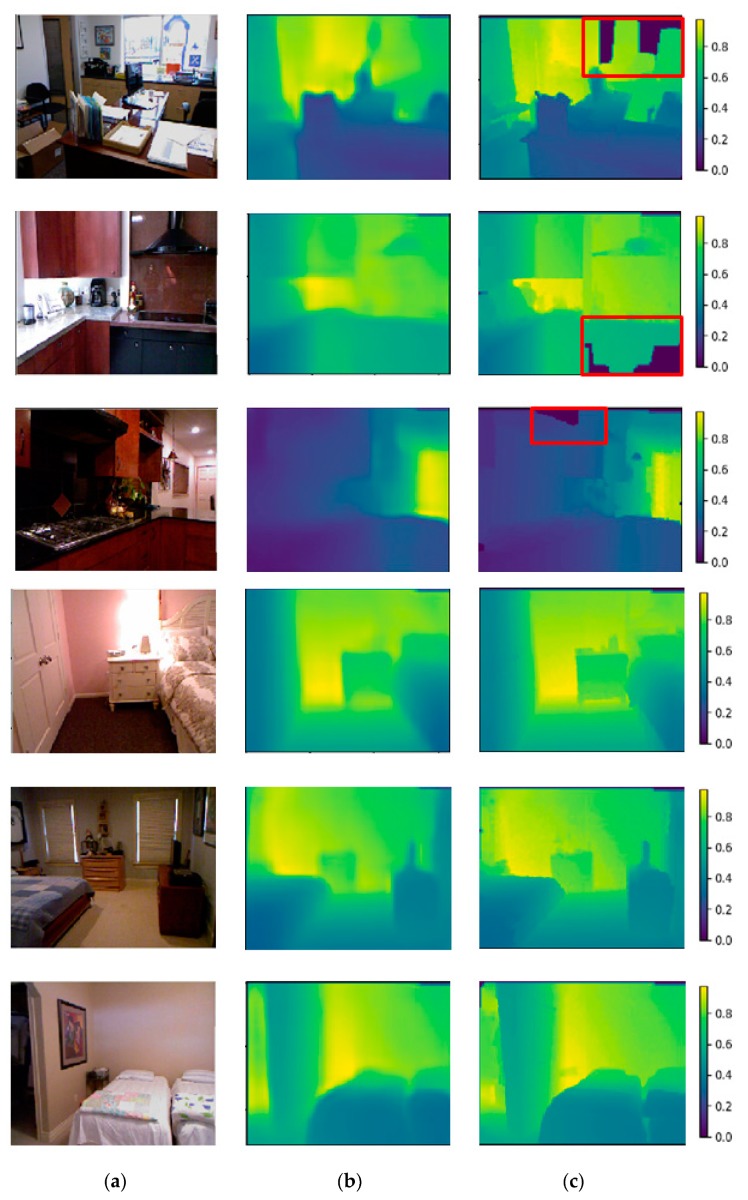
Example predictions from our algorithm. For each image we show (**a**) the input RGB image, (**b**) the prediction by our network, (**c**) the ground truth. The red border marks the area in the raw depth map where the depth map is missed. At the far right, the value of the scale labels from small to large represents the depth of the image (**a**) from near to far.

We trained the network for 120 K training data using a stochastic gradient descent (SGD) optimizer with batches of size 16 and we initialized the second stream with the ResNet-50 weights pretrained on Image-Net [37]. The initialization of other layers was done in accordance with the method officially recommended in version 0.4 of pytorch. In this paper, the three streams we designed are parallel training. In addition, different learning rates are set at different layers of the networks: 0.001 for the first streamed all layers and the third streamed convolutional layers 1 and 3, and 0.01 for the third streamed convolutional layers 2, 3 and 4. The starting learning rate is 0.01 for the other layers and gradually decreases. The momentum is 0.9 and the weight decay coefficient is 0.0001. Overall, the training time is approximately 70 h using a single NVidia TITAN X. We know that some depth images missed a few values in the raw NYU Depth v2 dataset, a result of shadows caused by the disparity between the infrared emitter and the camera or random missing or spurious values caused by specular or low albedo surfaces. However, our model can also make a better prediction of these images with some missing depth values. A few examples of our prediction with different inputs are displayed in Figure 3.

### 4.2. Baselines and Comparisons

Since the depth map size of our model output was 160 × 128, which was lower than the original images in the resolution, we upsampled the output to 640 × 480 by bilinear interpolation and compared it with the ground truth. We evaluated the performance of our method and compared it with previous work on the 654 NYU Depth v2 [18] image dataset using the same evaluation criterion as [25,26,30]. There are several categories:

- Threshold: % of $p_{i}$ s.t. $\max\left(\frac{p_{i}^{*}}{p_{i}},\frac{p_{i}}{p_{i}^{*}}\right)=\delta <threshold,$
- Mean relative error (rel): $\frac{1}{n}\sum_{p_{i}\in n}\frac{\left|p_{i}^{*}-p_{i}\right|}{p_{i}^{*}},$
- Mean $Log_{10}$ Error (log_10_): $\frac{1}{n}\sum_{p_{i}\in n}\left|\log_{10}p_{i}^{*}-\log_{10}p_{i}\right|,$
- Root mean squared error(rms): $\sqrt{\frac{1}{n}\sum_{p_{i}\in n}{\left(p_{i}^{*}-p_{i}\right)}^{2}},$
- Root mean squared error(rms(log)): $\sqrt{\frac{1}{n}\sum_{p_{i}\in n}{\left(\log_{10}p_{i}^{*}-\log_{10}p_{i}\right)}^{2}}.$

where $p^{*}$ is the ground-truth depth and p is the predicted depth, both also have an index *i*, *n* is the number of pixels in the test set and *threshold* is a constant: 1.25, 1.25^2^ or 1.25^3^.

**Figure 4:**
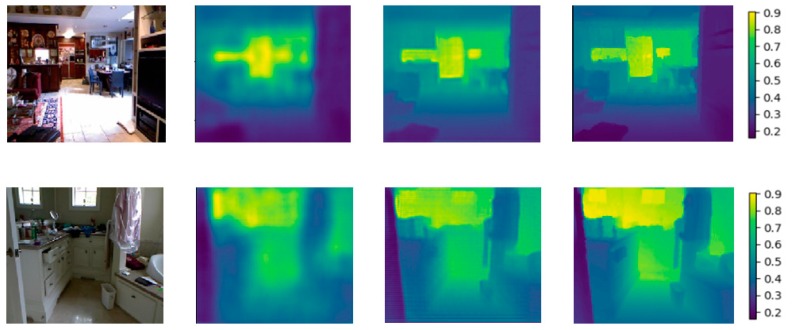

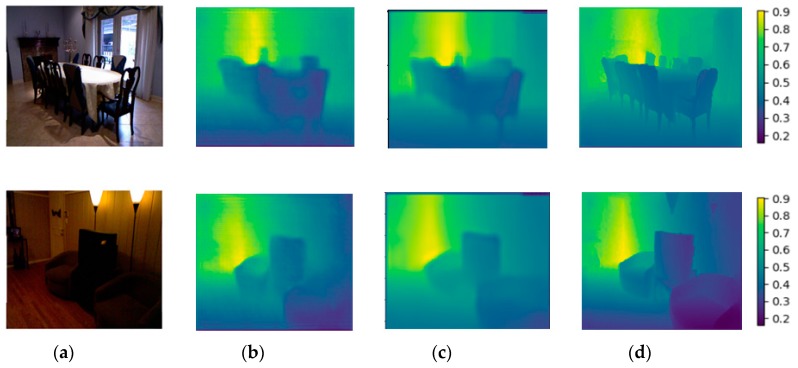
Example depth results. (**a**) RGB image; (**b**) result in Reference [30]; (**c**) our result; (**d**) ground truth. Note that the color range of each image represents the distance of the camera to the object.

To evaluate the effectiveness of our model we designed some experiments on the NYU Depth v2 dataset [18]. The most advanced results are almost all achieved by the full CNN, so we show the visual results of the depth map by our method and other methods in Reference [30]. The results are shown in Figure 4. Table 2 shows a quantitative comparison of the proposed model with the relevant work [19,20,25,26,27,30,31,33]. We can see from the results that our method outperforms competing methods for visual quality and other quality metrics.

As can be seen from the results of Table 2, the evaluation criteria of the proposed method are superior to other supervised learning methods. Our results are significantly improved compared with the traditional method [19,20] and compared with the method using CNNs [25,27,31], which is also superior to that in Reference [30]. We provided some examples of depth maps estimated by the method in Reference [30] and our method. Obviously, our method is very accurate even at the boundary of the object and the contour and details of the object are clearer than that of Reference [30]. Our method can also detect the regions with missing depth values.

## 5. Discussion and Conclusion

Predicting depth from a single RGB image is a challenging task. In this paper a prediction method based on PTSN is introduced. There are three novel contributions. First, we proposed pyramidal-structure images as the network input, which allows the extraction of multiscale features to improve the robustness of the model. Second, we defined a set loss function to train our network and achieve better accuracy than previous work. Finally, the small convolution kernel is used instead of a large kernel to realize an *upconvolution* structure; furthermore, the resolution of the output image is improved. Experimental results show that compared to other methods our method showed that the proposed network is able to exceed other techniques on this task for the NYU Depth v2 [18] datasets.

In the future, we will design a network of unsupervised learning to solve the problem of deep prediction and further verify on multiple data sets to improve the adaptability of our network. We will also apply the network to other useful applications, such as 3D SLAM, motion estimation or semantic segmentation.

## Figures and Tables

**Table 1:** Pyramidal third-streamed network (PTSN) architectures for the NYU Depth v2 [18] dataset.

Stream	Input	Block 1	Block 2	Block 3	Block 4	Block 5	Output
	160 × 120	3 × 3 conv	3 × 3 conv	3 × 3 conv	3 × 3 conv	3 × 3 conv	10 × 8
		64 channel	2 × 2 pool	2 × 2 pool	2 × 2 pool	2 × 2 pool	
First			64 channel	128 channel	256 channel	512 channel	
						0.5 dropout	
						(10,8) upsample	
Second	320 × 240		base network is resnet-50				10 × 8
Third	640 × 480	11 × 11 conv	5 × 5 conv	5 × 5 conv	5 × 5 conv	3 × 4 conv	10 × 8
		2 × 2 pool	2 × 2 pool	2 × 2 pool	0.5 dropout		

**Table 2:** Quantitative comparison with state-of-the-art-based methods on the NYU Depth v2 dataset [18]. For the δ accuracies, higher is better; for the others, lower is better.

Method	δ < 1.25	δ < 1.25^2^	δ < 1.25^3^	rel	log_10_	rms	rms(log)
Karsch et al. [19]	-	-	-	0.35	0.131	1.2	-
Liu et al. [20]	-	-	-	0.335	0.127	1.06	-
Li et al. [31]	0.621	0.886	0.968	0.232	0.094	0.821	-
Liu et al. [27]	0.650	0.906	0.976	0.213	0.087	0.759	-
Eigen et al. [25]	0.611	0.887	0.971	0.215	-	0.907	0.285
Eigen and Fergus et al. [26]	0.769	0.950	0.988	0.158	-	0.641	0.214
Chakrabari et al. [33]	0.806	0.958	0.987	0.149	-	0.620	-
Laina et al. [30]	0.811	0.953	0.988	0.127	0.055	0.573	0.195
ours	**0.818**	**0.958**	**0.988**	**0.123**	**0.053**	**0.569**	**0.189**
	**higher is better**			**lower is better**			
